# Bristol Girls Dance Project Feasibility Trial: outcome and process evaluation results

**DOI:** 10.1186/1479-5868-9-83

**Published:** 2012-07-02

**Authors:** Russell Jago, Simon J Sebire, Ashley R Cooper, Anne M Haase, Jane Powell, Laura Davis, Jade McNeill, Alan A Montgomery

**Affiliations:** 1 Centre for Exercise, Nutrition & Health Sciences, School for Policy Studies, University of Bristol, Bristol, UK; 2 Faculty of Health and Life Sciences, University of the West of England, Glenside Campus, Stapleton, Bristol, BS16 1DD, UK; 3 School of Social and Community Medicine, University of Bristol, Bristol, UK

## Abstract

**Background:**

Many adolescent girls do not engage in sufficient physical activity (PA). This study examined the feasibility of conducting a cluster randomized controlled trial (RCT) to evaluate an after-school dance program to increase PA among 11–12 year old girls in Bristol, UK.

**Methods:**

Three-arm, cluster RCT. Three secondary schools were assigned to intervention arm. Intervention participants received a 9-week dance program with 2, 90-minute dance classes per week. Participants at 2 control schools received incentives for data collection. Participants at 2 additional control schools received incentives and a delayed dance workshop. Accelerometer data were collected at baseline (time 0), during the last week of the dance program (time 1) and 20 weeks after the start of the study (time 2). Weekly attendance, enjoyment and perceived exertion were assessed in intervention participants. Post-study qualitative work was conducted with intervention participants and personnel.

**Results:**

40.1% of girls provided consent to be in the study. The mean number of girls attending at least one dance session per week ranged from 15.4 to 25.9. There was greater number of participants for whom accelerometer data were collected in control arms. The mean attendance was 13.3 sessions (maximum = 18). Perceived exertion ratings indicated that the girls did not find the sessions challenging. The dance teachers reported that the program content would benefit from revisions including less creative task time, a broader range of dance genres and improved behavioral management policies. At time 2, the 95% confidence intervals suggest between 5 and 12 minutes more weekday MVPA in the intervention group compared with the control incentives only group, and between 6 minutes fewer and 1 minute more compared with the control incentives plus workshop group. Between 14 and 24 schools would be required to detect a difference of 10 minutes in mean weekday MVPA between intervention and control groups.

**Conclusions:**

It is possible to recruit 11–12 year old girls to participate in an after-school dance study. An after-school dance intervention has potential to positively affect the PA levels of 11–12 year old girls but an adequately powered RCT is required to test this intervention approach.

## Background

Physical activity (PA) is associated with lower body mass, lipid and blood pressure levels among youth [1]. Many children do not meet the current UK recommendation [2] of an hour of moderate-to-vigorous intensity PA (MVPA) every day [3]. Physical activity levels decline during childhood [4]. Girls are less active than boys at all ages [4] and although the evidence base is increasing there is still a particular shortage of interventions to increase PA among girls [5,6].

The majority of adolescents regularly attend school and schools have structures that can facilitate the delivery of interventions [7]. Most of the school-based interventions that have focused on increasing PA have attempted to integrate provision during curriculum time but these changes have largely been ineffective [5]. One possible reason for this failure is the limits on the time during the school day that can be devoted to PA [8]. As such, extra-curricular programs that utilize the benefits of the school infrastructure, but do not impinge on curriculum time, could be an effective way to increase the PA levels of adolescent girls [9,10].

Dance is the favorite form of PA for UK girls [11] and many girls would attend an after-school dance program [12]. Girls who would normally drop out of most other forms of PA will engage in dance if it is available to them [13]. A recent paper has reported that adolescent girls obtain approximately 10 minutes of MVPA from hour long dance classes held at dance studios, but it is not clear how much MVPA could be obtained from dance classes provided to non-dancers as part of after-school provision [14]. Increasing the provision of dance within the extra-curricular program could be an effective means of increasing PA among large numbers of girls.

It is essential that intervention efficacy is established before dissemination [15]. A full examination of the effect of an after-school dance program intervention on the PA levels of secondary school aged girls would require a cluster randomized controlled trial (RCT). Cluster RCTs are very expensive to conduct, and therefore before proceeding to a full trial it is important to ensure the intervention is feasible and that the study is sufficiently powered to detect a change in the target behavior [15]. It is also important to identify at the piloting stage any aspects of the intervention that could be improved before progressing to a full trial.

In light of the information presented above, the overall goal of this study was to examine the feasibility of conducting a cluster RCT examination of an after-school dance program that is designed to increase PA among 11–12 year old girls. To address this goal we had six interlinked aims: 1) To assess the feasibility of recruiting and retaining 11-12 year old girls in an after-school dance program; 2) To examine the feasibility of collecting accelerometer data and examine the PA levels of consenting girls; 3) To examine the potential change in MVPA as a result of participating in the study; 4) To conduct a process evaluation of the intervention to examine factors that might be important for a full trial including session attendance, perceived exertion and perceived enjoyment; 5) To conduct post-intervention qualitative work to identify any refinements that could be made before proceeding to a full-trial; and 6) To provide the necessary information to calculate the sample size for a cluster RCT evaluation of an after-school dance program.

## Methods

### Design and recruitment

The Bristol Girls Dance Project (BGDP) was a three-arm, parallel group, cluster randomized controlled pilot trial, with schools as the unit of allocation. Seven secondary schools were recruited from the three school districts that form the greater Bristol area. We asked the Dance Specialist responsible for coordination of dance provision in each school district to identify all of the schools within their district without current after-school dance provision for Year 7 girls (8, 6 and 4 schools in Districts A, B and C respectively). Eligible schools were asked to participate in a research study to examine the potential utility of an after-school dance program.

All Year 7 (11–12 years old) girls who were physically able to participate in physical education classes were invited to participate in the study. In the intervention schools, a “taster session”, which was broadly representative of the intervention content, was delivered to all Year 7 girls. At the end of the session project staff provided study information including participants’ commitment to attend two sessions per week and the requirement to provide data at three time-points.

At the control schools, all Year 7 girls were asked to participate in a research study about PA and provide data at three time points. All control group participants were told at the briefing meeting whether they would receive incentives for data collection only or incentives and a dance workshop. Potential participants in all seven schools were told that there was a maximum of 30 randomly assigned spaces (see below) in the project in each school. The study was approved by a University of Bristol ethics committee and informed parental consent was obtained for all participants.

### Randomization

The seven participating schools were randomly allocated to the three arms (described below), stratified by school district. Randomization was conducted by a member of a clinical trials unit with no other involvement in the study, using computer-generated random sequences and anonymised codes for school district and school name.

### Description of intervention and control groups

The three intervention schools received two, 90-minute after-school dance classes per week for 9 weeks. The 9-week duration was selected to allow the entire program to be delivered within the Spring school term. For practical reasons the sample was limited to a maximum of 30 girls per school. The dance class content included opportunities for participant input and time to practice short dance pieces [12]. All sessions were based on the hip-hop/street dance genre. At the end of the intervention all participants were provided with information about local dance opportunities. Instructors were provided with the outline dance program and attended a half-day content familiarization session.

As our pilot work had suggested that dance is a very attractive form of PA for girls [12], we wanted to ascertain whether offering a dance workshop at the end of the research process (i.e., after the last data collection), would affect either retention or the quality of data provided by participants. We therefore utilized a three-arm design with two different control groups. In two schools, participants were provided with small thank you gifts of £3, £5 and £10 for data collections 1, 2 and 3 respectively (“Control incentives only” group). In the other two schools participants were provided with the same small thank you gifts as well as a half-day dance workshop at the end of the study (“Control incentives + workshop” group).

### Measures

#### Aims 1 to 3: Feasibility of recruiting girls and collecting accelerometer data

Counts and proportions of the number of girls in the school providing parental consent were used to address aim one. In order to assess the feasibility of collecting accelerometer data (Aim 2) and the likely change in MVPA that could be expected from taking part in the program (Aim 3) all participants were asked to wear an Actigraph accelerometer (Model GT1M; ActiGraph LLC, FL, USA) at baseline (Week 0), during the last two weeks of the intervention (Week 8 or 9) and 3 months after the intervention had ended (Week 20). The accelerometers were worn for seven days and were set to record data every 10 seconds. Periods of ≥60 minutes of zero values were defined as accelerometer “non-wear” time and discarded. Participants were included in the analysis if they provided ≥3 weekdays of data with at least 500 minutes of data between 6 am and 11 pm. A cut-point of ≥2296 counts per minute [16] was used to identify mean minutes of weekday MVPA and mean minutes of MVPA in the weekday after-school period (3-6 pm).

To establish whether the participants were similar to a larger sample of Year 7 girls from the Bristol area, baseline data were compared to data from Year 7 girls sampled in the PEACH Project. PEACH (Personal and Environmental Associations with Children’s Health), is a survey of 1300 children from Bristol who were assessed in their final year of primary school and again one-year later in their first year of secondary school [17,18].

#### Aim 4: Process evaluation

Attendance was recorded at each session by the dance instructor. During weeks 3–7 (i.e. once the intervention was established and before the final assessments), attending participants completed a modified version of the OMNI perceived exertion scale [19] (1–10 scale) once per week. Participants were also asked to complete a brief enjoyment (1–5 scale) survey that had been developed for PE lessons [20] and used successfully in a Pilates intervention for primary school girls [21], once a week at the end of the dance session. At the time 2 assessment intervention participants were asked if they were currently attending dance programs.

#### Aim 5: Post study qualitative work

Following the intervention, semi-structured interviews were held with the three dance instructors (mean duration = 44.6 minutes). Six semi-structured focus groups (two per intervention school) were conducted with 6–7 girls in each group (mean duration of 44.5 minutes). The focus group participants were purposively sampled to include pupils with a range of intervention attendance levels within each school. Both the interviews and focus groups focused on the elements of the program that could be improved.

### Analyses

#### Quantitative data - aims 1 to 4 and 6

We used appropriate descriptive statistics (frequencies, percentages, means, standard deviations) to describe the process measures of recruitment, attendance and retention in the study. To facilitate comparisons these descriptive data were also calculated for the PEACH sample. We used linear regression models to compare differences in means and 95% CI between the trial groups at follow up for the PA variables, adjusted for baseline PA, area stratification and the clustering of participants within schools. As the data are from a feasibility trial and we are not powered to detect a difference between groups p values are not reported. We estimated potential sample sizes for a future trial (Aim 6) using different combinations of key parameters (i.e., type I and type II error levels) within the SAMPSI command. These calculations were based on the assumption that a future trial would have a single intervention and a single control arm. The calculations also assumed that a ten minute per day between-group difference in mean MVPA per day would be the smallest effect size worth detecting. Ten minutes more MVPA per day would represent an increase of 50 extra minutes of MVPA per week and equate to a 1/3 increase on baseline levels. (Evidence shown below also indicates that this difference would be achievable with the current intervention). All analyses were performed using Stata 11 (Statacorp, College Station, Texas).

#### Qualitative data - aim 5

All focus group and interview recordings were transcribed verbatim and anonymised. As the data are considered exploratory, we adopted a thematic analytical approach. Using NVivo (Version 8, QSR, Southport, UK), meaningful content was coded and codes were grouped to form themes that described the content of codes [22]. Quotes that were deemed to best represent the nature of each theme were then extracted. All codes and emergent themes were checked by an independent researcher and discussed to ensure consistency of coding and resolve any discrepancies.

## Results

The recruitment, consent and baseline data provision rates are shown by school in Table 1. The mean consent rate across all seven schools was 40.1%. Baseline accelerometer data were collected from 203 (97%) of the 210 participants enrolled. The comparison of BMI and PA data between study participants and Year 7 girls from the PEACH Project indicated that the mean BMI of the two groups were comparable (19.1 vs. 19.9 kg/m^2^) for the BGDP and PEACH girls respectively. However, the BGDP participants engaged in 19.5 fewer minutes of MVPA per day (33.2 vs. 52.3). (Data not in Tabular form).

**Table 1:** Recruitment, consent rate, data provision and weekly attendance in the Bristol Girls Dance Project

School	Arm allocation^**a**^	Number of girls in school	Provided consent (n, %)	Enrolled in intervention (n, %)	Provided baseline data (n, %)		Mean weekly attendance during intervention (n, %)
					Questionnaire	Accelerometer^b^	
1	A	106	43 (40.6)	30 (28.3)	30 (100)	28 (93.3)	15.4 (51.3)
2	A	100	43 (43.0)	30 (30.0)	30 (100)	29 (96.7)	24.8 (82.6)
3	A	112	42 (37.5)	30 (26.8)	30 (100)	30 (100)	25.9 (86.3)
4	B	112	57 (50.9)	-	30 (100)	30 (100)	-
5	C	83	44 (53.0)	-	30 (100)	29 (96.7)	-
6	C	101	41 (40.6)	-	30 (100)	27 (90)	-
7	B	179	48 (26.8)	-	30 (100)	30 (100)	-

^**a**^ A **=** Intervention, B = control + incentives, C = control + incentives + workshop; ^b^ Numbers represent provision of valid data.

Table 2 shows descriptive, anthropomorphic and PA data at baseline in each trial arm. Mean weekday minutes of MVPA ranged from 34.3 (standard deviation (SD) = 17.3) in the control incentives + workshop group to 40.2 (SD = 18.1) minutes per day in the control incentives only group.

**Table 2:** Means and standard deviations (SD) for anthropomorphic and physical activity data by trial arm at time 0 (baseline)

	**Intervention**		**Control (incentives)**		**Control (incentives + workshop)**	
	***Mean***	***SD***	***Mean***	***SD***	***Mean***	***SD***
Height (m)	1.52	0.07	1.51	0.08	1.51	0.08
Weight (kg)	44.53	8.49	43.86	10.14	43.60	8.57
BMI (kg/m^2^)	19.10	2.87	18.95	3.14	19.08	3.00
BMI SDS	0.35	1.02	0.26	1.13	0.33	1.08
MVPA/weekday (mins)	35.95	16.59	40.21	18.11	34.33	17.27
MVPA afterschool (mins)*	12.08	7.66	15.99	8.53	12.98	7.13

* MVPA recorded between 3 pm and 6 pm on a school.

The provision of accelerometer data is presented by trial arm in Table 3. The percentage of accelerometer data provided at time 1 was greater in the two control arms that received data collection incentives than in the intervention arm (88-93% vs. 80%). These differences were more marked at the time 2 assessment when 92% of the control incentives only group provided accelerometer data with a much lower figure of 68% in the intervention group.

**Table 3:** Percentage of randomized participants per trial arm providing overall accelerometer data at Time 0, 1 and 2

		**Intervention**			**Control**			**Control**			**Total**		
					**(incentives)**			**(incentives + workshop)**					
		*N*	*%*	95% CI	*N*	*%*	95% CI	*N*	*%*	95% CI	*N*	*%*	95% CI
Time 0	Valid	87	96.7	0.93 to 1.00	60	100.0	-	56	93.3	0.87 to .99	203	96.7	0.94 to 0.99
	Invalid	3	3.3	0.00 to 0.07	0	0	-	4	6.7	0.00 to 0.13	7	3.3	0.01 to 0.06
	Missing	0	0	-	0	0	-	0	0	-	0	0	-
Time 1	Valid	72	80.0	0.72 to 0.88	56	93.3	0.87 to 1.00	53	88.3	0.80 to 0.97	181	86.2	0.82 to 0.91
	Invalid	10	11.1	0.05 to 0.18	1	1.6	0.02 to 0.05	5	8.3	0.01 to 0.16	16	7.6	0.04 to 0.11
	Missing	8	8.9	0.03 to 0.15	3	5.0	0.01 to 0.11	2	3.3	0.01 to 0.08	13	6.2	0.03 to 0.09
Time 2	Valid	61	67.8	0.58 to 0.78	55	91.7	0.84 to 0.99	46	76.7	0.66 to 0.88	162	77.1	0.71 to 0.83
	Invalid	11	12.2	0.05 to 0.19	3	5.0	0.00 to 0.99	3	5.0	0.01 to 0.11	17	8.1	0.04 to 0.12
	Missing	18	20.0	0.12 to 0.28	2	3.3	0.01 to 0.08	11	18.3	0.08 to 0.28	31	14.7	0.10 to 0.20

Valid = participant’s accelerometer data met the wear time criteria.

Invalid = participant’s accelerometer data did not meet the wear time criteria.

Missing = participant did not provide accelerometer data.

**Figure 1:**
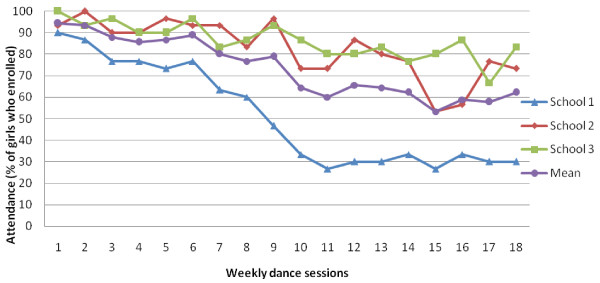
Attendance per dance session expressed as percent of who enrolled presented by school and overall.

Figure 1 presents the average attendance levels for the intervention schools. Average attendance declined from a little over 90% at session 1 to around 60% at the final session. The mean was heavily influenced by attendance at school 1, which was markedly lower than the two other schools. The mean overall number of sessions attended was 13.29 (SD = 5.12) out of 18 possible sessions. This figure also differed by school with the girls at school 1 attending half of the sessions (9.0, SD = 5.7) but better attendance at school 2 (15.0, SD = 2.6) and school 3 (15.5, SD = 4.0).

**Figure 2:**
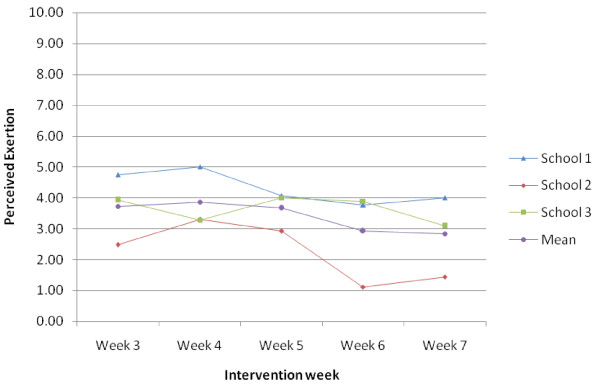
Mean perceived exertion ratings (1–10 scale) for weeks 3–7 by school and overall.

**Figure 3:**
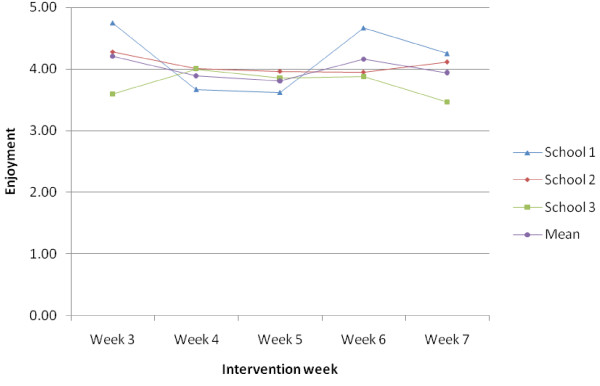
Mean enjoyment ratings (1–5 scale) for weeks 3–7 by school and overall.

The mean perceived exertion and enjoyment ratings are shown by school and intervention week in Figure 2. The mean perceived enjoyment ratings were approximately 4 on a 1–5 scale indicating good levels of enjoyment across the intervention weeks (Figure 3). At the time 2 assessment, 41 of the 76 intervention girls (53.9%) who provided data reported still attending some form of dance classes.

Differences in PA between the intervention group and the two control groups are presented in Table 4. At time 1, the 95% confidence intervals suggest between 18 minutes fewer and 4 minutes more weekday MVPA in the intervention group compared with the control incentives only group, and between 8 minutes fewer and 3 minutes more compared with the control incentives + workshop group. At time 2, the 95% confidence intervals suggest between 5 and 12 minutes more weekday MVPA in the intervention group compared with the control incentives only group, and between 6 minutes fewer and 1 minute more compared with the control incentives + workshop group. Similar patterns were evident in a comparison of the mean after-school MVPA minutes per day between the groups.

**Table 4:** Physical activity data by trial arm and adjusted between group differences at time 1 (8 weeks) and 2 (20 weeks)

	**Control (incentives)**			**Intervention**			**Control (incentives + workshop)**	
	**Time 1**							
	M	SD	I vs CI adjusted difference in means (95% CI) ^†^	M	SD	I vs CIW adjusted difference in means (95% CI) ^†^	M	SD
**MVPA/weekday (mins)**	48.7	21.5	−6.8 (−17.9 to 4.1)	38.8	19.1	−2.2 (−7.8 to 3.5)	39.6	21.1
MVPA/day after school (mins)*	16.7	8.6	−0.11 (−5.6 to 5.4)	14.6	8.7	1.0 (−3.3 to 5.3)	13.0	10.0
	**Time 2**							
**MVPA/weekday (mins)**	49.7	18.4	8.7 (5.5 to 11.9)	51.4	18.7	−2.4 (−5.7 to 0.9)	51.8	20.6
MVPA/day after school (mins)*	14.7	6.3	2.2 (0.9 to 3.5)	14.3	6.4	−2.3 (−5.2 to 0.5)	17.6	8.3

^†^ Between group differences always compare to the intervention arm and are adjusted for baseline outcome scores & area stratification.

MVPA = moderate-to-vigorous physical activity.

* After school period = 3–6 PM.

Table 5 shows sample sizes for a potential trial powered to detect a difference of 10 minutes of MVPA per weekday (i.e., 50 minutes per week) between an intervention and control group. We derived the school-associated intra-class correlation (0.018) for weekday MVPA at time 2 (95% CI = <0.001 to 0.087), and used the upper 95% confidence limit in our sample size estimates. Depending on specified power and alpha, 14 to 24 schools would be required, assuming a cluster size for analysis (after attrition), of 24 participants per school.

**Table 5:** Sample size calculations based on detecting a 10-minute differences in weekday MVPA and two arm trial

**Outcome**^**a**^	**ICC**^**b**^	**α**	**Power (β)**	**N required**	**N inflated for attrition**	**n schools/arm**	**Total n schools**^d^
MVPA/weekday (min)	0.087	5%	80%	236	295	7	14
MVPA/weekday (min)	0.087	5%	90%	314	393	9	18
MVPA/weekday (min)	0.087	1%	90%	440	550	12	24

^a^Target difference = 10 minutes of MVPA.

^b^ observed ICC (95% CI) was 0.018 (<0.001 to 0.087).

^c^ estimated attrition = 20%.

^d^ based on 24 pupils per school.

### Post-study qualitative data

#### Dance teacher interviews

Overall the dance teachers were positive about the intervention, but they did suggest some ways in which the intervention content could be improved including a broader dance focus that changed dance styles during the intervention.

"“If you were doing it again, maybe look at other styles of dance.” (Dance Instructor 2)"

"“Maybe at the start of the project go through a few different styles and then play with them a bit.” (Dance Instructor 1)"

A key element of the intervention was the creative tasks that were provided by the instructors to facilitate pupil autonomy and ownership. The dance teachers thought that these elements had merit but found implementing them challenging within their groups.

"“Sometimes it was really hard to get them all doing it, with only one of me and thirty of them all in pairs or groups… as soon as I turned my back, one group would just sit and so it could be quite difficult to manage.” (Dance Instructor 2)"

Generally, the instructors described the participants’ behavior as good. However, at times, all instructors encountered behavioral problems with their groups including lateness and arguments during group work. The instructors were unsure of how to address these problems within the boundaries of the project.

"“I wasn’t sure whether I could say, “you’re not welcome to come back any more”, because their behavior wasn’t changing over a certain amount of weeks. And so it was empty threats.” (Dance Instructor 2)"

"“The lateness and behavioral question…maybe it needs a conversation with someone like (School Contact) so I know what their behavioral systems are in schools and whether I can carry any of those over.” (Dance Instructor 3)."

### Student focus groups

Overall the girls were very positive about the dance program and found the sessions to be enjoyable.

"“I found it quite funny at first; it was really new so it was different from what we usually do. You really wanted to do it more”. (School 3, participant 5)"

"“I think it was good that everyone could get involved in stuff, even if you haven’t done any dance before”. (School 2, participant 3)"

The girls welcomed having the opportunity to provide input into the sessions and were generally supportive of the elements of the program that provided time for creative input.

"“Yeah I thought it was quite good that we got to do what we wanted a bit as well, so yeah, choose what we could do.” (School 3, participant 5)"

"“I like working in partners, doing your own little dances”. (School 2, participant 2)"

The girls suggested that the dance sessions did not adapt in intensity as their perceived fitness levels increased.

"“I thought on the first and second sessions I thought ‘wow this is really tiring and I’m going to have to really work hard,’ but then I got used to it and it became a bit easier”. (School 3, participant 2)"

"“I didn’t think it was too bad… I don’t think that was too tiring or anything”. (School 2, participant 5)"

"“I don't think it was very challenging”. (School 3, participant 13)"

## Discussion

The overall goal of this study was to examine the feasibility of conducting a cluster RCT of an after-school dance program that is designed to increase the PA levels of 11–12 year old girls. The data presented here demonstrate that it is feasible to recruit participants into the study, as on average 40% of the girls within the schools expressed an interest in taking part. Further, it was possible to retain participants’ engagement in both intervention and control arms and collect data from a majority of them at three time points. These findings support previous school-based health intervention research [23]. Interestingly, there did not appear to be any difference in terms of recruitment or data provision rates between schools that were offered a delayed dance workshop and those who were not. This suggests that it is not necessary to provide a delayed dance workshop to enhance retention in the trial.

The provision of accelerometer data was higher in both the control arms than the intervention arm at both of the follow-up assessments. Participants in both control arms were provided with small incentives in order to facilitate data collection but the intervention group was not provided with these incentives. As such, the data appear to suggest that these relatively small incentives were sufficient in order to facilitate the provision of data. The findings therefore suggest that if we were to repeat the study there would be merit in providing small incentives for data collection to all participants.

The comparison between the participants included in this study and the girls from the PEACH project [17,18] indicates that on average our sample was less active than the PEACH girls. Previous studies have suggested that dance engages girls less likely to take part in organized team sports in PA [11,13] and our data appear to support this hypothesis.

The attendance data indicate that on average more than 60% of the girls who enrolled attended the program. Mean attendance levels in the current study were attenuated by the attendance levels at school 1. The causes of these differences are not immediately clear and it is hard to judge the extent to which these differences mirror expected intra-school variability. It is however, noticeable that it was the dance teacher in school 1 who most strongly expressed concerns about her relationship with the school in the process evaluation. As preliminary research has suggested that school climate might be associated with girls’ PA [24], it is possible that the school climate had an effect on the study participants in each school. It would therefore be useful to assess school climate if a larger evaluation of the intervention were to be conducted.

On average the girls reported a mean perceived exertion level of five or lower. In a previous pilot study it was reported that the mean perceived exertion rating recorded by 11 year old girls during Pilates, an activity which would normally be considered to be a lower intensity than dance, was 5.9 on a comparable 1–10 scale [21]. The BGDP student focus groups suggested the perceived intensity of the dance sessions decreased over the duration of the intervention, indicating that the session intensity did not increase in line with improvements in fitness. The dance teacher interviews suggested that the creative tasks were not always an effective use of time with many girls becoming distracted during these periods. The dance teachers also indicated that broadening the content of the sessions might facilitate skill progression. Collectively, data indicate that in a full trial the program would benefit from some refinement in which the PA duration and intensity are increased by reducing the non-active time during creative tasks and broadening the type of dance. It is notable that the plateau in exertion and increase in enjoyment seen from week five onwards in school 1 coincides with a plateau in attendance following a sharp decline. This could reflect retention of the most motivated and enthusiastic participants in the later stages of the intervention in this school who provided greater enjoyment and exertion ratings.

Interpreting the likely effect of the intervention on MVPA is difficult both because there was a small sample size, which makes it impossible to adequately test for differences in means, and because the change patterns were not consistent across the two control arms. Comparing the intervention arm to the control incentive only arm at time 2 indicated a difference of 8.7 minutes of weekday MVPA per day but wide confidence intervals (5.5 to 11.9). However, when comparing the intervention group to the control group which received incentives for data collection and a delayed dance workshop, weekday MVPA levels were actually lower in the intervention group by 2.4 minutes with 95% confidence intervals that ranged from −5.7 to 0.9. These findings are somewhat hard to reconcile, as it is unlikely that the offer of a delayed dance program would account for this difference. There was a difference in the number of participants providing valid accelerometer data at the time 2 assessments with levels of 67.8%, 91.7% and 76.7% obtained among the intervention, control incentives and control incentives plus workshop groups respectively. It is therefore possible that these findings and the wide confidence intervals are a function of the small sample size. When viewed in the round, these findings support a full adequately powered trial to determine whether an after-school dance program has the potential to increase PA, but as noted above, some refinement of the intervention may be necessary.

The sample size calculations suggested that based on 24 children in each school for analysis we would need to have 393 children or 18 schools to detect an average increase of 10 minutes per day in MVPA on a weekday with 90% power and an alpha of 0.05. We used a cluster size of 30 children per school (24 for analysis after attrition), as discussions with schools and dance teachers indicate that this class size is the maximum that could be handled by a single dance teacher. A 10 minute per day weekday increase in MVPA would equate to an extra 50 minutes of MVPA per week and represent an increase of approximately 30% on baseline PA. A ten minute per weekday increase would be within the 95% confidence intervals for the difference between the intervention and incentive only group comparisons at time 2 and would compare favorably with previous PA interventions that have focused on increasing PA provision in the after-school period. For example, in a recent review of after-school PA interventions, Pate and O’Neill reported that three of the five randomized controlled trials that had been conducted had reported positive increases in objectively measured PA [10]. Of particular interest is the Stanford Sports to prevent Obesity Trial (SPORT) which reported a ten minute increase in moderate intensity PA after provision of an extracurricular soccer program [25]. Thus, a ten minute per weekday increase in MVPA would represent a marked increase in PA levels and is consistent with successful PA interventions.

### Strengths and limitations

The major strengths of this study are the detailed collection of recruitment, attendance and data provision quality in a relatively large feasibility trial which can inform the design of a future intervention. It is however, important to recognize that the data reported here originate from a feasibility trial that was not powered to detect differences between groups. It is also important to acknowledge that only 30 girls per school took part and as such not the entire year group of girls received a benefit from participating in the intervention. It is also important to recognize that although the evidence suggests that the taster session encouraged less active participants to sign up for the intervention, the taster may also have discouraged some girls from joining the program. As such, the sample may be oriented towards girls who liked the taster session. However, as noted above it appears that the PA levels of the girls who participated in the study are lower than average activity levels in this age group and that as such the sample represented “low active” girls.

## Conclusions

In this study we have shown that it is possible to recruit 11–12 year girls to participate in an after-school dance intervention and the associated control arms. We have also shown that girls will attend the dance sessions and that it is feasible to collect PA data from the girls at three time points but providing small incentives facilitates greater data provision. The data demonstrate that after-school dance programs have potential to positively affect the PA of 11–12 year old girls, but a larger trial would be required to fully test this hypothesis. Using the data collected in this study we estimate that a sample of 393 girls, recruited from 18 schools would be needed to detect a 10 minute per weekday difference in MVPA. After-school dance is an intervention that holds potential as a means of increasing 11–12 year old girls PA, but a larger trial is needed to fully assess this possibility.

## Competing interests

We have no competing interests to declare.

## Authors’ contributions

The study was conceived by RJ, ARC, AAM, AMH and JP. LD was the project manager and data were collected by LD, JM and SJS. The analysis plan was conceived by RJ, AAM and SJS. Analysis was performed by SJS and supervised by AAM. RJ drafted the first version of the manuscript with additional sections provided by all authors. All authors provided critical edits and revisions to the paper and have reviewed and approved the final version of the paper.
